# Nanocarrier drug resistant tumor interactions: novel approaches to fight drug resistance in cancer

**DOI:** 10.20517/cdr.2020.81

**Published:** 2021-06-19

**Authors:** Aleksandra Benko, David Medina-Cruz, Ada Vernet-Crua, Catherine P. O’Connell, Małgorzata Świętek, Hamed Barabadi, Muthupandian Saravanan, Thomas J. Webster

**Affiliations:** ^1^AGH University of Science and Technology, Faculty of Materials Science and Ceramics, Krakow 30059, Poland.; ^2^Department of Chemical Engineering, Northeastern University, Boston, MA 02115, USA.; ^3^Institute of Macromolecular Chemistry, Czech Academy of Sciences, Prague 16206, Czech Republic.; ^4^Department of Pharmaceutical Biotechnology, School of Pharmacy, Shahid Beheshti University of Medical Sciences, Tehran 19919-53381, Iran.; ^5^Department of Medical Microbiology and Immunology, Division of Biomedical Sciences, School of Medicine, College of Health Sciences, Mekelle University, Mekelle 231, Ethiopia.; ^*^These authors contributed equally to this work.

**Keywords:** Drug-resistance, cancer, nanotechnology, nanocarriers, drug delivery

## Abstract

Cancer is one of the biggest healthcare concerns in our century, a disease whose treatment has become even more difficult following reports of drug-resistant tumors. When this happens, chemotherapy treatments fail or decrease in efficiency, leading to catastrophic consequences to the patient. This discovery, along with the fact that drug resistance limits the efficacy of current treatments, has led to a new wave of discovery for new methods of treatment. The use of nanomedicine has been widely studied in current years as a way to effectively fight drug resistance in cancer. Research in the area of cancer nanotechnology over the past decades has led to tremendous advancement in the synthesis of tailored nanoparticles with targeting ligands that can successfully attach to chemotherapy-resistant cancer by preferentially accumulating within the tumor region through means of active and passive targeting. Consequently, these approaches can reduce the off-target accumulation of their payload and lead to reduced cytotoxicity and better targeting. This review explores some categories of nanocarriers that have been used in the treatment of drug-resistant cancers, including polymeric, viral, lipid-based, metal-based, carbon-based, and magnetic nanocarriers, opening the door for an exciting field of discovery that holds tremendous promise in the treatment of these tumors.

## Drug-resistant tumors

Cancer comprises a conjunction of different diseases, which involve the uncontrollable growth of cells generating tumors. Cancerous cells can interfere with various cell processes, such as attachment, division, or motility. Additionally, they can spread into other tissues, forming metastases^[1]^. Nowadays, cancer is one of the major healthcare concerns worldwide. According to the World Health Organization, approximately 10 million people died in 2018 around the globe due to cancer. Moreover, in the US, cancer is the second leading cause of death, with over half a million deaths per year, and the American Cancer Society estimates that there will be approximately 2 million new cases diagnosed by 2020^[2]^.

The battle to fight cancer has been present since early human civilization. Reports from the Ancient Greeks and Romans describe basic surgeries to remove breast tumors. However, it was not until the 1940s when chemotherapy treatments were implemented, using drugs to reduce or eliminate cancer cells^[3]^. Since then, a wide variety of treatments combining physics, biology, and molecular genetics have been used to improve recovery rates, such as radiotherapy, hyperthermia, or immunotherapy. Nonetheless, there is still not a definitive cure, and most treatments rely on chemotherapy combined with radiotherapy used to sensitize cancer cells. This increases chemotherapy efficacy while causing increased systemic toxicity and mortality^[4,5]^.

**Figure 1:**
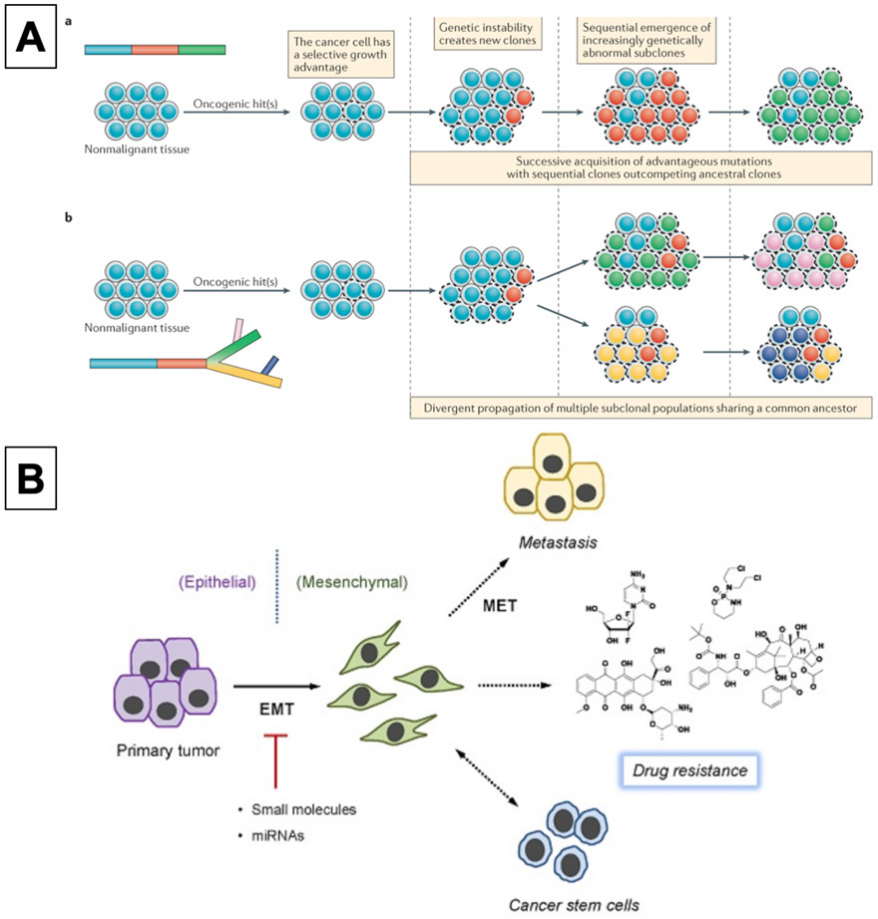
Tumor heterogeneity evolution (a) linear or (b) branched (A)^[20]^; Epithelial-mesenchymal transition mechanism (B)^[23]^

Drug-resistance in cancer is the ability of cancerous cells to become tolerant of the treatments that once could kill them. There are multiple factors responsible for developing resistance; some rely on evolution and spontaneous mutations (intrinsic), others are a consequence of the uptake of drugs (acquired or extrinsic)^[6]^. Over time, leading mechanisms of drug resistance were described as drug inactivation, drug target alteration, drug efflux, reduced drug uptake, cell death inhibition, and DNA damage repair [Figure 1]^[7]^. Approximately 90% of current chemotherapy treatment failures are related to drug resistance, indicating the seriousness of this issue^[8]^.

Drug inactivation is a consequence of the interaction between the drug and the proteins present in the body, which can affect its structure and/or the molecular mechanisms of the drug. For instance, overexpression of tau, a microtubular-associated protein, was reported to participate in docetaxel (DTX) resistance in various prostate cancer cells^[9]^. Moreover, as some types of anti-cancer drugs are activated upon metabolic digestion, disturbance of this process leads to incomplete activation and acquisition of drug resistance^[10]^. Another mechanism of drug resistance is known as the alteration of drug targets. Modifications and mutations can affect either the drug target or the signaling pathway it controls, which can alter drug efficacy^[11]^. For instance, different mutations in the epidermal growth factor receptor (EGFR) and tyrosine kinase inhibitors (TKI), a target for osimertinib in lung cancer, resulted in an increased drug-resistance. Suzawa *et al.*^[12]^ specifically showed how the amplification in the MET gene could account for up to 22% of resistance in the EGFR and TKI.

Drug efflux is one of the most studied mechanisms of drug resistance in cancer. The ATP-binding cassette (ABC) family are a group of transport proteins that move compounds such as amino acids, ions, lipids, and drugs through the cell membrane. An alteration or overexpression in the cell transporters can affect the drug export, uptake, and accumulation, making chemotherapy drugs unusable. Specifically, drug efflux is known as the process of drug pumping outside the cell^[13]^. Interestingly, this process may be used to develop resistance against one drug while causing sensitivity towards another. This was proven by Hermawan *et al.*^[14]^, who found that continuous treatment with salinomycin induced drug resistance against salinomycin, while treatment sensitized drug-resistant cancer cells towards DOX.

There are two main processes of cell death - autophagy and apoptosis - which are known as programmed necrosis and cell death, respectively. These processes can be modified or inhibited, inducing cancer cell resistance. Apoptosis resistance can be acquired by different pathways, via receptors, like the tumor necrosis factor family or mitochondria. Meanwhile, autophagy is mainly related to hypoxic processes^[15]^. For instance, alterations in the programmed cell death ligand (PD-L1) cause the acquisition of resistance in anaplastic lymphoma kinase proteins of non-small cell lung cancer^[16]^. On the other hand, Toth *et al.*^[17]^ showed two different resistance pathways for patients with bone-metastatic castration-resistant prostate cancer. *In vivo* and *in vitro* modeling demonstrated that resistance to phosphoinositide 3-kinase (PI3K) induced reduced oxidative stress and prevented cell death in a hypoxic microtumor environment.

Moreover, some chemotherapy drugs, especially platinum-based ones, have demonstrated the ability to cause either indirect or direct DNA damage, which can trigger resistance. DNA damage repair systems, such as the recombination repair system or the nucleotide excision repair system, are complexes whose principal function is to amend the damage. In cancer cells that develop resistance, those DNA damage repair systems can be inhibited by mutation or gene silencing, leading to further mutations and increasing resistance^[18]^. As described by Nogales *et al.*^[19]^, the presence of several enzymes such as DNA and RNA helicases in ovarian and lung cancer was related to Pt/carboplatin resistance, and gene silencing was reported to prevent the drug resistance-associated to platinum-derived compounds.

Tumor heterogeneity and microenvironment can also play an important role in drug-resistance. Tumors can have wide variability in terms of phenotype and morphology, such as differential gene expression and motility or metastatic potential. Motility and metastatic potentials are related to epigenetic, transcriptomic, genetic, and proteomic factors such as translocations of RNA or chromosomal rearrangements^[20]^. This variability can be seen in cells coming from the same tumor type; therefore, each cancer cell can react differently when subjected to the same drug. As a consequence, lesion-specific responses were described in some cancer types such as colorectal, when targeted therapies were used^[21]^. Specific extracellular matrix growth factors and stromal cells found in the tumor microenvironment have also been reported to participate in drug-resistant mechanisms as they interfere and modify cell communication^[22]^.

Furthermore, the epithelial-mesenchymal transition (EMT) mechanism is an emerging area of research in terms of cancer drug resistance. EMT is a biological process in which tumors become metastatic. A suppression or modification in EMT can induce changes in cell adhesion and attachment receptors, as well as cell motility and angiogenesis processes^[23]^. EMT, other than promoting the formation of more metastatic cells, can also enhance the survival of those already present, leading to unwanted drug resistance to the treatments^[24]^. As an example, the pre-mRNA processing factor (PRPF), a protein from the kinase family, was found to be overexpressed in some cancers such as prostate and melanoma. In that case, due to arrangements in actin in the cytoskeleton, alterations in cellular morphology occurred. It was demonstrated that PRPF triggers EMT, blocking the apoptotic effects of some drugs like resveratrol, hence triggering drug-resistance in HCT116 colon cancer cells^[25]^. Furthermore, the formation of persister cancer cells (PCC), rare immortal cells that can self-renew through division, is connected with the EMT effect. PCC is in the quiescence state, mitigating traditional therapies that target fast-proliferating cells. These cells are also thought to be responsible for cancer remission and are particularly hard to target^[26]^.

In summary, drug resistance in cancer cells limits the efficacy of current approaches to defeat cancer, especially the late-diagnosed, metastatic ones, decreasing the curability and survival rate and increasing the risk of disease recurrence and relapse. Although several mechanisms such as drug efflux, drug target inactivation, tumor heterogeneities, or DNA damage repair are extensively studied, there are still plenty of unknowns in the field and a large need to develop new treatment strategies. In this review, we will revise and discuss several technologies based on nanomedicine approaches to overcome cancer cell resistance and analyze future challenges that need to be addressed.

## Nanocarriers for the treatment of drug resistance in tumors

Nanomaterials have been extensively investigated for their use in anticancer therapies to improve therapeutic approaches such as drug delivery, increase the efficacy of treatment, reduce side effects, and overcome drug resistance. The use of nanomaterials for drug delivery to tumors has been presented as an alternative to conventional chemotherapy treatments over the past years. An interesting advantage of nanoparticle-assisted drug delivery is the selective targeting to tumors, which might overcome several factors, such as dose-limiting side effects, lack of selectivity, tissue toxicity, limited drug access to tumor tissues, reduction of effective drug doses, and the emergence of multiple drug resistance with conventional or combination chemotherapy^[27]^.

Nanoparticles (NPs) can be designed in a wide range of sizes, shapes, and compositions to be used for passive or active targeting of cancerous cells. Throughout the years, different strategies of drug delivery have been elucidated, including active targeting where NPs directly interact with cells or bind with specific receptors or antibodies expressed on the cells. Alternatively, the use of passive targeting is based on the enhanced permeability (EPR) effect. The EPR effect is the ability of molecules of 100-1000 nm in size to accumulate in the leaky vasculature exhibited on tumor tissue^[27]^.

Once the loaded NPs accumulate in the desired target, they can release the drug through different stimuli, such as temperature or pH change, respond to enzymes or antigens, or physical stimuli. Physical stimuli can include a magnetic field, light, or heat. This approach is gathering considerable research interest due to its higher versatility^[28]^.

**Figure 2:**
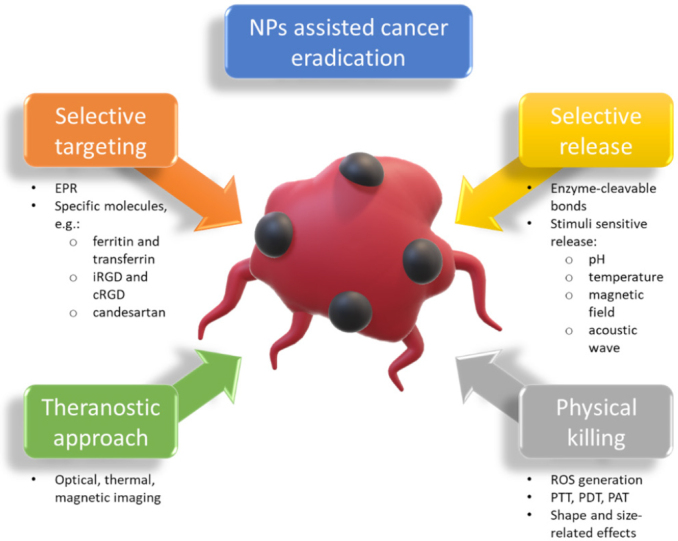
A graphical representation of NP-assisted cancer-killing effects. NP: nanoparticle; EPR: enhanced permeability

Over time, more and more nanostructures have been discovered and designed, and their applicability in the field of nanomedicine has been extensively studied. Currently, different nanomaterials and nanoformulations, such as polymeric lipid and metal NPs, as well as nanovesicles (e.g., dendrimers and liposomes), have emerged as innovative, effective, and promising platforms for the treatment of drug-resistant cancer cells^[29]^. Multiple benefits and mechanisms of NP-specific cancer-killing effects have been identified, and these are presented in Figure 2.

A few NP-drug formulations are already FDA approved, e.g., Abraxane (albumin NPs + PTX), Genexol-PM (polymeric micelles + PTX), and Doxil (liposome + DOX). These NPs formulations provide better results than conventional drug treatments^[30]^. Nanocarriers have been reported to improve the therapeutic drug index, introduce the ability for multifunctional treatments, divert the ABC-transporter mediated drug efflux mechanism and selectively target the tumor cells, cancer stem cells, tumor-initiating cells, or cancer microenvironment^[31]^.

In the following sections, different nanocarriers will be explored for their effectiveness in fighting the progression of cancer-resistant tumors.

### Polymeric nanocarriers

#### Polymeric micelles

**Figure 3:**
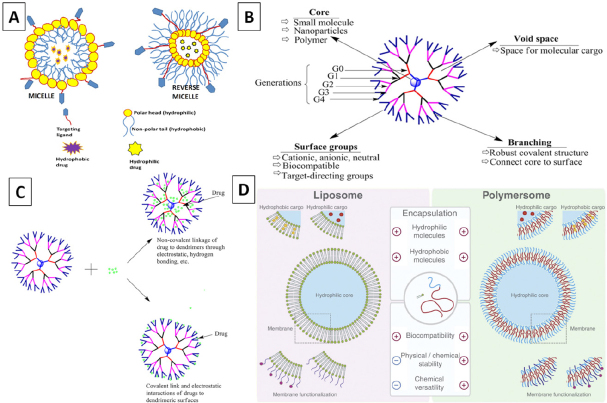
Schematic representation of polymeric micelle and reverse polymeric micelle (Original figure) (A); Schematic representation of a dendrimer (B)^[38]^; Schematic representation of possible types of drug-dendrimer interactions (C)^[38]^; Schematic representation of liposomes (left) versus polymersomes (right) (D)^[47]^

Polymeric micelles (PM) are colloidal particles of a self-assembling nature. They have multiple interaction points compared to conventional micelles, and their structure comprises of two parts: the hydrophilic head and the hydrophobic tail^[32]^. Figure 3A represents a schematic illustration of PM and reverse PM that can be utilized for drug delivery systems.

Tumor targeting with the help of micelles can be achieved by ligand-mediated targeting (folate, carbohydrate, proteins, peptides, and monoclonal antibodies), internal stimuli-responsive PM (pH-responsive PM, enzyme-responsive PM, and thermoresponsive PM), and external stimuli-responsive PM (magnetic field-responsive PM, light-responsive PM, and ultrasound-responsive PM)^[33]^.

Moreover, micelles can be made of many different polymers and biopolymers, all of which were proven effective in targeting metastatic or chemo-resistant cancers. For example, chitosan PM, a biopolymeric structure, was coupled with stearic acid-containing DOX and injected intravenously in breast tumor-bearing BALB/c mice to study the uptake of the drug by the circulating monocytes. Almost 94% of the circulating monocytes took up the injected PM, which was visualized with the help of fluorescent molecules. Then, it was exocytosed by macrophages and taken up by 4T1 cancer cells, which shows the efficacy of the treatment method^[34]^.

Alternatively, synthetic polymers, PEG, and its derivatives are most commonly used to form PM. Due to their high versatility, good biocompatibility, and ease of modification, PEG-based PMs have proven to be useful in the selective targeting of drug-resistant cancers. Yao *et al.*^[35]^ have developed PEG-based polymeric micelles, which were cleaved by matrix metalloproteinase-2 (MMP2), the enzyme involved in cancer progression and metastasis, for selective release of PM’s payload (DOX and dasatinib) in cancer cells. The formulated micelles showed significant cytotoxicity against drug-resistant NCI/ADR-RES and MES-SA/Dx5 cell lines. The authors confirmed that the formulated micelles could effectively inhibit the drug efflux caused by dasatinib and improve drug efficacy in MDR cells with DOX when used as a co-administered efflux inhibitor or as a micellar drug nanocarrier^[35]^.

Another example of PM-assisted targeting drug-resistant cancer can be found in a study by Gao *et al.*^[26]^ Herein, PEG-based PMs were used to promote ferroptosis in PCC cells. Two separate factors were used to induce ferroptosis: conjugation of arachidonic acid (ferroptosis precursor) and encapsulation of RSL3, glutathione peroxidase 4 (GPX4) inhibitor, arresting lipidic repair. The drug-carrier was postulated to accumulate in cells via the EPR effect and selectively release its cargo in reactive oxygen species (ROS)-rich cancer environments. As a result, a 30-fold increase in human ovarian adenocarcinoma cells’ killing efficiency was observed *in vitro* and as compared with control micelles. *In vivo*, selective accumulation in cancer and effective payload release resulted in improved survival rates, connected with tumor growth inhibition, and no adverse side effects were observed^[26]^.

An example of multifunctional PM can be found in the study by Zhen *et al.*^[36]^, in which PEG-based PMs [polymeric prodrug (PMP)] were encapsulated with novel NIR fluorophore (DEB-BDTO) as a photosensitizer along with a drug efflux pump inhibitor - TQR and PTX. As a result, an efficient PDT enabling drug carrier was fabricated, which selectively releases a chemotherapeutic drug and resistance development inhibitor upon NIR irradiation. The formulated micelles showed a prominent synergistic lethal effect when paired with photodynamic therapy and chemotherapy against resistant SKOV-3/MDR, in both *in vitro* and *in vivo* mice models. Tumor growth inhibition was observed for up to 17 days, and no adverse side-effects were identified^[36]^. In conclusion, the studies mentioned above suggest that careful tailoring of the PM compositions can be used to reverse the drug resistance and effectively target tumors.

#### Dendrimers

Dendrimers are nano-sized, 3D highly branched compounds^[37]^. They comprise three major parts: a central core, repeating units of continuously branched dendrons, and functional groups. Functional groups are located at the periphery of the dendrimer where modifications can be made, and the drug compounds can be attached to the dendrimer^[38]^. Figure 3B represents a schematic illustration of a dendrimer that can be utilized for drug delivery systems, and Figure 3C illustrates possible types of drug-dendrimer interactions.

Dendrimers are generally employed for *in vitro* diagnostics, drug and vaccine delivery, gene transfection, and protein mimicking^[39]^. Common dendritic molecules used in the drug delivery applications are polyamidoamine (PAMAM), polypropylene imine (PPI), poly-L-lysine (PLL), polyglycerol, and polyglycerol-co-succinic acid. Of the dendrimers listed above, PAMAM and PLL are the most commonly used. The great advantage of using dendrimers lies in their good biocompatibility, combined with extremely high drug loading capacity^[40]^ and excellent delivery efficiency, which allows deep penetration of the drug into the tumor^[41]^. Stable systemic circulation and selective release of the drug at the tumor vasculature can be obtained by fabricating multi-component materials^[42,43]^. Also, multi-component materials can be combined with PDT therapy^[44]^.

As for targeting drug-resistant cancers, dendrimers were found to provide excellent outcomes. Pan *et al.*^[45]^ formulated a PAMAM dendrimer with a PEG copolymer. When this dendrimer was co-loaded with DOX and therapeutic siRNA (siMDR-1), the formulated material showed significant anticancer efficacy against MDR human ovarian carcinoma (A2780/ADR) and breast cancer (MCF7/ADR). The authors confirmed the downregulation of P-glycoprotein in treated cells, which led to overcoming the resistance towards DOX^[45]^. Additionally, Gouveia *et al.*^[46]^ formulated polyalkylidenimine dendrimers functionalized with the organometallic moiety [Ru(η^5^-C_5_H_5_)(PPh_3_)_2_]^+^. The system showed significant anticancer efficacy against a cisplatin-resistant human ovarian carcinoma (A2780cisR) cell line.

#### Polymersomes

Polymersomes are a type of drug vehicle that have an aqueous interior and bilayer chambers that can carry both hydrophilic and hydrophobic drug molecules. These nanocarriers are synthetic analogs of liposomes and are made of amphiphilic block copolymer membranes [Figure 3]^[47]^. Polymersomes are usually copolymerized with PEG, and the formed copolymer can be linked to the hydrophilic, hydrophobic, or biological blocks for efficient drug delivery applications^[48]^. Usual block compositions are polybutadiene-block-ethylene oxide (PB-b-PEO)^[49]^, polycaprolactone (PEG-b-PCL)^[50]^, and folic acid-poly-L-glutamic acid-block-poly-ε-caprolactone [FA-PGA-b-PCL]^[51]^.

Impressively, polymersomes exhibit greater versatility and stability than liposomes, with properties easily steered by the composition and molecular weight of co-polymers^[47,52]^. They are biodegradable so that they can help in the sustained release of the desired drug molecule in the biological environment^[53]^. Also, they have already been successfully employed in carrying certain anti-cancer drugs like PTX for enhanced intraperitoneal chemotherapy, GEM for lung cancer, and DTX for breast cancer^[54-56]^.

For instance, polymersomes were successfully used for combinational chemotherapy, where PTX and DOX were employed for treating hepatocellular carcinoma. The hydrophobic core of the polymersomes contained hydrophobic drugs: PTX and DOX, while the outer surface was formed by a dense, hydrophilic PEG-folate corona. Such composition allowed for good dispersibility and selective targeting of cancer cells through folate receptors. The particles were readily and selectively up-taken by BEL-7404 cells *in vivo*, followed by a sustained pH-responsive release of the drug. As a result, tumor growth was slowed down by 80%, with no adverse side effects observed^[52]^.

Qin *et al.*^[57]^ formulated a folate-decorated a triblock copolymer PCL_7500_-ss-PEG_7500_-ss-PCL_7500_ based redox-responsive polymersome, which was loaded with TQR, DOX, and PTX. The formulated polymersome exhibited elevated drug accumulation into MCF-7/ADR cells via the TQR-induced P-glycoprotein efflux inhibition, and the cell cycle was blocked in the G2/M phase.

#### Summary

Polymeric nanocarriers are a wide group of materials whose qualities can be easily tailored for meeting specific needs. Specifically, the time of degradation and size can be highly fine-tuned, and this is particularly useful for ensuring EPR and controlled release. However, their intrinsic properties do not allow for easy employment of a theranostic approach and combining multiple killing mechanisms to effectively fight chemoresistant or metastatic cancers. These features can be introduced through further modifications, which can be troublesome and hard to design. In particular, it is hard to ensure multiple binding mechanisms for different additives, and competing reactions can, therefore, be expected. For these reasons, examples of multifunctional polymeric nanocarriers are rather scarce in the literature, mostly concerning encapsulation of more than one additive. Still, there are some examples of excellent studies that have undertaken the goal of combining multiple actions in elegant, carefully designed carriers, and these are studies by Gao^[26]^ and Zhen^[36]^. High cancer-killing efficiency, selectivity, and ability to completely reverse the chemoresistance prove that such an approach should be treated as guidance for further development of polymeric nanoparticles. In the future, more strategies that would allow the introduction of a theranostic approach should also be pursued.

### Viral nanocarriers

**Figure 4:**
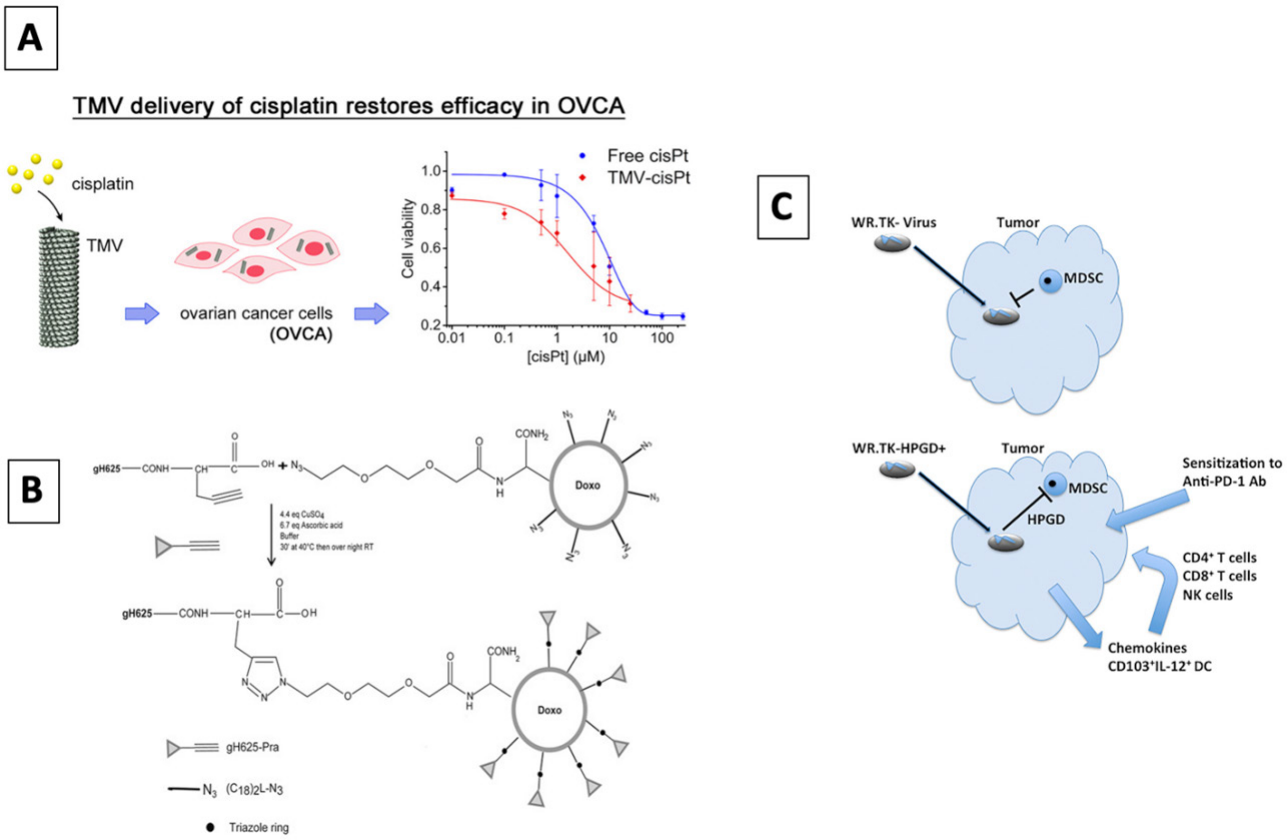
Tobacco mosaic virus (TMV) delivery of Pt in platinum-resistant ovarian cancer cells (A)^[58]^; DOX delivery in gH625 Herpes virus derived-protein encapsulated liposome in drug-resistant lung adenocarcinoma cells (B)^[59]^; Western reverse (WR) vaccinia targeting PGE2 to overcome immunotherapy resistant cancer cells (C)^[61]^

Viral nanocarriers, also known as viral-based nanoparticles (VNPs) or virus-like particles (VLPs), are virus-based materials that present a nanometric size with a specific geometry [Figure 4A]. They are called viral nanocarriers as they are constructed with viruses or viral proteins acting as nanocages; hence, their potential to be used as drug delivery carriers, imaging, gene therapy, or vaccination purposes. VNPs are highly versatile, allowing multiple morphological and surface chemistry modifications while being highly biocompatible^[31]^.

VNPs have demonstrated their potential to overcome possible resistance to chemotherapy drugs. For instance, VNPs made by tobacco mosaic virus (TMV) loaded with Pt showed enhanced efficacy towards both platinum-resistant and platinum-sensitive ovarian cancer cells. The encapsulation of the drug within the viral structure allowed for increased efficacy and overall lower dosage with superior cytotoxicity and DNA-double strand breakage *in vitro*^[58]^. The use of virus-derived proteins has also shown efficacy against chemotherapy-resistant tumors. The reason behind this is related to the ability of these proteins to cross the cellular membrane, conferring a directed drug-delivery character to the therapy. As an example, a gH625 protein, derived from Herpes simplex virus 1 (HSV-1), was conjugated to DOX (DOX) and encapsulated into a liposome [Figure 4B]. The as-received system was able to induce apoptosis in the DOX-resistant lung adenocarcinoma cell line. The authors demonstrated that it was the presence of the viral particle, which allowed the enhanced delivery to overcome the drug resistance^[59]^.

Moreover, oncolytic viruses (OV), VNPs that are based on living viruses, can provide unique cancer-fighting strategies that are characterized as immunotherapy since they can replicate inside the tumor, allowing them to spread along with the tissue. OV proliferate inside the tumor using multiple pathways, inducing immunogenic cell death without causing any effect on healthy tissue^[60]^. For that reason, OV is a rising interest in the cancer drug resistance field. The use of oncolytic vaccinia has been studied in recent years. Patients generally present highly variable immune defects and suppression, which lead to increased cancer resistance. Recent investigations show that molecules such as prostaglandin E2 (PGE2) play vital roles in cancer cell resistance.

Some viral vectors can target PGE2 and reverse localized immunosuppression. In their research, Hou *et al.*^[61]^ used a modified-Western Reverse strain of vaccinia virus against multiple immunotherapy-resistant cancers in mice. The viral formulation expressed hydroxyprostaglandin (HPGD), which successfully targeted PGE2 and depleted granulocytic myeloid-derived suppressor cells (G-MDSC) to overcome resistance. Other studies indicated that the endoplasmic reticulum (ER) stress response could reprogram resistance in some cancer cell lines using oncolytic viruses like Maraba rhabdovirus. The main aim of the study was to sensitize cancer cells to OV and make the therapy more efficient and effective using a small molecule inhibitor called IRE1α. Glioblastoma tumors undergo an enhanced caspase-derived apoptotic cell death when in contact with the viral agent, as tested in murine models^[62]^.

Vesicular stomatitis virus (VSV) is par excellence in the prototypic OV. Nonetheless, some studies have shown an increased cancer cell resistance to it. Ongoing investigations suggest that the use of some inhibitors can be successfully applied to reverse this effect in prostate, ovarian, or glioblastoma cancer. Upon silencing histone deacetylase (SIRT1), prostate cancer cells become sensitive to the virus and allow its replication and spread^[63]^.

Similarly, the use of interferon modulators in a VSV modified with a glycoprotein from lymphocytic choriomeningitis virus (GP) allowed the VSV to overcome partial resistance in ovarian cancer cells. The combination of GP-VSV with the inhibitor ruxolitinib showed enhanced cytotoxicity towards ovarian cancer cell lines without damage to healthy tissues or signs of resistance or remission^[64]^. Similar types of modulators derived from different kinds of viruses, e.g., the Semliki Forest virus, were previously applied successfully in glioblastoma^[65]^.

#### Summary

The use of VNPs or OVs to fight chemoresistant cancers is a relatively new approach that has not yet been well studied. Still, it seems to be very promising, and multiple studies report efficient eradication of cancer cells, while the main advantage lies in VNPs’ ability to easily and selectively penetrate the cells. Some of the cited studies report excellent efficiency in cancer eradication *in vivo* upon intratumoral^[61,64]^ or intraperitoneal^[62,65]^ injections in tumor-bearing mice models. The treatment was able to either arrest the tumor growth^[61,62,64]^ or eradicate it^[65]^. What is more, VNPs or OVs can provide long-term immunity against tumor regrowth. Nonetheless, there is still a lot of investigation to cover to apply these techniques, such as: understanding the tumor heterogeneities, analyzing the *in vivo* toxicities, and analyzing long-term effects. Specifically, in the cited studies, severe side effects in animal models were reported, with viral escape and transfection of healthy tissues observed, followed by neurological symptoms. Such reactions should be properly identified, and mitigating steps need to be designed before the carriers can be regarded as safe and be qualified for further studies. One must also take into account that VNPs or OVs alone will not allow for employing combinatorial approaches and instead should be treated as an addition to other strategies. For example, they could be loaded inside a certain carrier along with other compounds, thus permitting theranostic and/or multiple cancer cell killing mechanisms.

### Lipid nanocarriers

#### Liposomes

**Figure 5:**
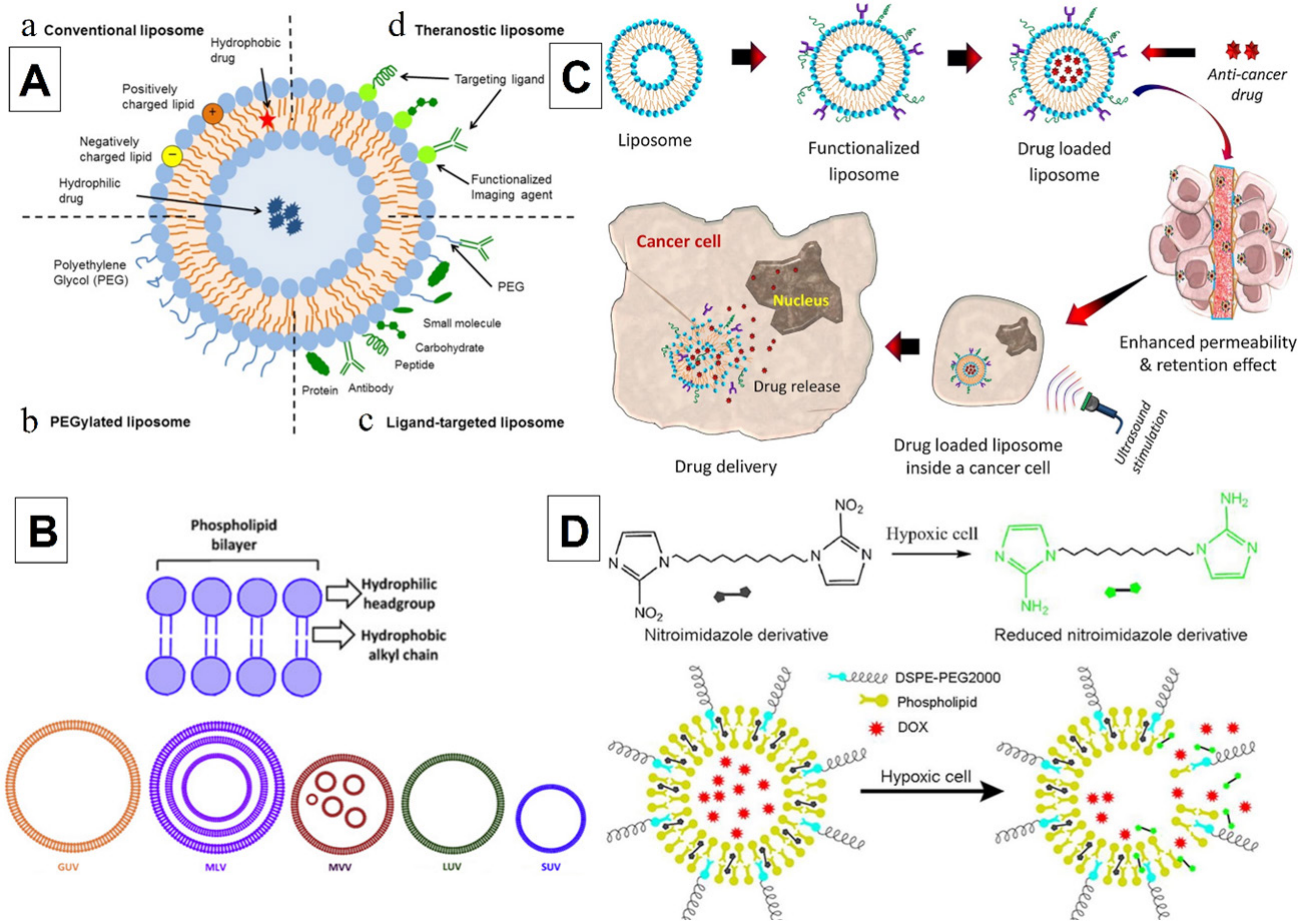
Schematic representation of the different types of liposomal drug delivery systems. Conventional liposome (a), PEGylated liposome (b), Ligand-targeted liposome (c), Theragnostic liposome (d)^[72]^ (A); Schematic representation of the different types of liposomes^[67]^ (B); Schematic representation of liposome-based drug delivery system for cancer therapy^[68]^ (C); Schematic illustration for triggered release of hypoxia-responsive liposomal drug delivery system (D)^[196]^

Liposomes are spherical phospholipid vesicles consisting of one or more phospholipid bilayers enclosing an aqueous core and are biomimetic to natural biological membranes^[66-69]^. Liposomes are biodegradable, biocompatible, nonimmunogenic, and possess a high loading capacity. With their amphiphilic nature, liposomes can deliver both hydrophilic (inside the core) and hydrophobic (embedded inside the phospholipid bilayer) drugs^[69-71]^. However, these particles have some shortcomings: instability, short blood circulation time, and insufficient drug loading capacity. To overcome these issues, PEGylated liposomes were developed, and these can be further modified with different therapeutic and diagnostic agents as well as antibodies, proteins, carbohydrates, vitamins, and glycoproteins for targeted drug delivery systems [Figure 5A]^[72]^.

Due to their excellent biocompatibility, liposomes were amongst the first studied cancer drug carriers, with some formulations already FDA-approved: Doxil/Caelyx® (pegylated liposomal DOX in the range of 100 nm), Marqibo® (vincristine sulfate liposome injection in the range of 100 nm), Onivyde® (IRI liposome injection in the range of 110 nm), Vyxeos® (liposomal cytarabine/daunorubicin in the range of 107 nm), and DaunoXome® (daunorubicin citrate liposome injection in the range of 40 to 80 nm)^[73]^.

Their usage is already well established, and recent efforts are focused on enhancing their selectivity, which is typically completed by using pH-sensitive systems, modifying the materials with cancer-specific molecules, or introducing additional molecules that can combat the chemoresistance. Some studies combine all three approaches. One example is a study by Paliwal *et al.*^[74]^, who reported that DOX-loaded hyaluronic acid-targeted pH-sensitive liposomes were more efficacious than free DOX after 48 h of treatment against CD44 receptor over-expressing MCF-7 cells with the IC_50_ values of 1.9 and 3.2 µmol/L, respectively.

Additionally, the *in vitro* release investigation revealed the pH-dependent release of DOX from the formulated drug-loaded liposomes, with faster release at mildly acidic pH ~5 in comparison to physiological pH ~7.4. *In vivo* performance was tested in skin tumor-bearing mice. The carrier was found to reduce the DOX accumulation in vital organs, revealing reduced systemic toxicity. At the same time, effective targeting of tumors resulted in its size reduction by 80% on the 30th day of experimentation^[74]^.

In a different study, Chen *et al.*^[75]^ formulated a pH-sensitive liposomal system in which positively charged liposomes (for easier cellular internalization) were covered with a negatively charged and pH-sensitive polymer (PEG-PLL-DMA), which was responsible for improving the drug carrier blood circulation time. The formulated materials showed a charge-reversal effect: they were negatively charged under a physiological pH value of 7.4, but the charge changed to positive in a pH value of 6.5 (in tumorous tissues). This was due to the cleavable amide linkages formed between the PEG-PLL and DMA. Positively charged carriers could then be easily internalized by cancer cells where the carriers could release cargo, specifically, NO donor diethylenetriamine diazeniumdiolate (DETA NONOate), for inhibiting the efflux pump and PTX, for cytotoxic effect against cancer cells. The *in vivo* studies on A549/T tumor-bearing mice (PTX resistant cells) revealed that the obtained system improved the drug’s accumulation and residency time inside the tumor. This leads to significant tumor necrosis and size reduction compared to a drug administered without a carrier. Additionally, it was observed that side effects towards vital organs were mild^[75]^.

Another strategy to fight drug-resistant cancer is to combine multiple drugs into one liposomal carrier. The most common method involves combining a traditional chemotherapeutic drug with another molecule, whose role is to reduce or mitigate resistance mechanisms. Among different compounds, two anti-malaria drugs, dihydroartemisinin (DHA) and chloroquine phosphate (CP), have been reported to participate in the process of cell sensitizing. The former has been suggested to inhibit the mTOR (mammalian target of rapamycin), responsible for pro-survival mechanisms in cells^[76]^, while the latter inhibits the efflux transporters^[77]^.

The therapeutic effect can then be further boosted by using cancer-selective molecules. In a study by Kang *et al.*^[78]^, a mannosylated liposomal tumor-targeting co-delivery system showed significant anticancer activity by combining DHA with DOX against drug-resistant human colon cancer HCT8/ADR cells overexpressing the mannose receptor. The IC_50_ value was 0.073 μg/mL, while free DOX or DHA did not show any cytotoxicity to this drug-resistant cancer cell line. *In vivo* studies in tumor-bearing mice revealed selective accumulation of the drug carrier in the tumor, leading to a nearly 90% tumor inhibition rate, as compared to 70% reported in combo drugs only. Furthermore, usage of the carrier was able to reduce the DOX-related side effects in the form of hepatic damage.

Meanwhile, Qiu *et al*.^[77]^ reported effective anticancer activity of a composite liposomal system co-encapsulating PTX with CP to treat PTX-resistant carcinoma. The authors confirmed that their synthesized liposomal system could block the efflux of PTX by ABC transporters. The authors proved that using multiple drugs in one system is far more effective than administering them in separate carriers. This was tested in an *in vivo* mice model, where tumor size inhibition was two times more significant than in the case of single or separately administered drugs, which was explained by a synchronized internalization pattern.

Other compounds that can be used to reduce the drug pumping in the liposome-based carriers out are coumarin, TGPS, or hydroxypropyl-β-cyclodextrin (HP-β-CD). In a study by Li *et al.*^[79]^, liposomes were co-loaded with coumarin and DOX and coated with TGPS, and their efficiency was tested against resistant A549/DDP cells, both *in vitro* and *in vivo*, as a xenograft tumor in the mice model. The materials were found to be readily up-taken by cancer cells, reducing their viability by up to 90%. The IC_50_ was nearly two times lower than in the commercially available drug, Taxotere®. *In vivo*, the tumor growth was almost completely arrested, with no adverse side effects observed. By comparing the system to the FDA-approved drug, the scientists were able to prove the excellence of their material.

Similarly, promising results were reported by Shen *et al.*^[80]^. In this study, liposomes were co-loaded with HP-β-CD and administered to fight PTX-resistant A549/T lung cancer cells, both *in vitro* and *in vivo* (mice model). With the IC_50_ reduced 2-fold as compared to free PTX and significantly enhanced apoptosis induction, the system was able to completely inhibit the tumor growth, with no adverse side effects observed.

While most drug carrier strategies are focused on improving the circulation time and cancer cell internalization while inhibiting the drug efflux mechanisms, some are even more fine-tuned. This approach can be found in a study by Li *et al.*^[81]^, in which the authors had decided to focus on improving the nuclear uptake of DOX. To do so, an AS1411 aptamer with high nucleolin binding affinity (overexpressed in the cancer cell nucleus) was complexed with DOX and loaded into the liposomes. The obtained materials were evaluated for their efficiency in eradicating drug-resistant breast cancer (MCF-7/ADR) cells *in vitro*. The authors confirmed that DOX-Ap liposomes bound with nucleolin strongly and eventually accumulated in the nuclei to effectively kill the MCF-7/ADR cancer cells. Compared to pure DOX, the IC_50_ value was reduced nearly 5-fold.

#### Solid lipid nanoparticles

**Figure 6:**
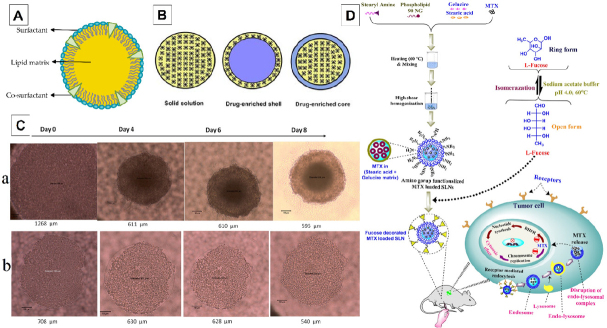
Schematic representation of the SLNs structure, representing the solid lipid matrix, surfactant, and co-surfactant^[83]^ (A); Classification of three types of classical SLNs including drug-enriched shell, drug-enriched core, and solid solution^[85]^ (B*);* Images of spheroids formation in different period of time with respective size (μm). MCF-7/ADR (a), NCI/ADR (b). Images are representative of triplicate samples^[93]^ *(*C); Development of fucose decorated methotrexate loaded SLNs (D). The proposed mechanism of action is methotrexate via ligand-receptor mediated endocytosis and internalization^[96]^

Solid lipid nanoparticles (SLNs) are an emerging generation of colloidal nanocarriers that consist of surfactant-stabilized biodegradable lipids based on a solid lipid matrix ranging from 50 to 1000 nm^[82]^. As shown in Figure 6A, the SLNs comprise the solid lipid matrix, surfactants, and occasionally co-surfactants^[83]^. Typically, waxes (e.g., cetyl palmitate), triglycerides (e.g., tripalmitin), steroids (e.g., cholesterol), fatty acids (e.g., decanoic acid), and partial glycerides (e.g., glyceryl behenate) are the most used lipids^[84]^. A drug-enriched shell, drug-enriched core, and solid solution (homogeneous matrix) are three possible models of SLNs-based drug carriers, depending on differences in the melting point between drugs and lipid matrices [Figure 6B]^[85]^.

The advantages of SLNs for drug delivery systems include the utilization of biocompatible and biodegradable ingredients, the lack of application of organic solvents, drug protection from environmental damage, high physical stability, ease of preparation, controlled drug release, and ability of CNS targeting^[86]^. SLNs have been successfully used to encapsulate lipophilic and hydrophilic medicines^[87]^, peptides/proteins^[88]^, and nucleic acids^[89]^. In recent years, the use of SLNs for anticancer drug delivery has become a rapidly growing field of study^[90,91]^.

When applying SLNs to fight drug-resistant cancers, usually a combination of more than one drug, the addition of a surfactant to inhibit the drug efflux pumping out or usage of specific cancer cell-targeting molecules was used. More advanced studies suggest a combination of either two or all three approaches. Affram *et al.*^[92]^ suggested a GEM-loaded SLN, fabricated from glyceryl monostearate (GMS), using two different surfactants in varying ratios: polysorbate 80 (Tween® 80) and poloxamer 188 (Pol 188). Herein, the surfactants were used to inhibit the drug efflux mechanisms. Among the twenty-four formulations tested in the study, the best one was found to be 1.0 (w/v%) GMS, 3.5 (w/v%) Tween 80, and 0.05 (w/v%) GEM with an average particle size of 603 ± 19 nm and entrapment efficacy of 68.3% ± 4.8%. This material exhibited higher cytotoxicity against the patient-derived primary pancreatic cancer cell line (PPCL-46) and human pancreatic Mia-PaCa-2 cells, compared to free GEM using monolayer (2D) and spheroid (3D) cell cultures. In 2D cultures, the IC_50_ values were reduced 3-fold for Mia-PaCa-2 and over 4.5-fold for the in PPCL-46, compared to pure GEM. In 3D cultures, these values were two times and four times lower, respectively.

In a more complex study, Oliveira *et al.*^[93]^ formulated SLNs, co-loaded with DOX, and α-Tocopherol succinate (TS) (DOX-TS-SLNs), with sizes in the range of 74 to 80 nm and entrapment efficacy of 99%. The aim was to obtain the synergistic effect, determine the ability to kill chemoresistant cells by disrupting their nuclei and mitochondria, and induce apoptosis. To improve internalization and reduce the drug efflux, materials were also coated with tocopheryl polyethylene glycol succinate (TPGS) as a surfactant. Efficiency was tested against drug-resistant cancer cells (breast MCF-7/ADR and ovarian NCI/ADR) growing in monolayer and spheroid cultures. The materials revealed significant cytotoxicity towards MCF-7/ADR and NCI/ADR cells. The cytotoxicity was higher when a combo drug was administered than when a single-drug loaded carrier was applied. In 2D cultures, the IC_50_ values were found to be 3-4-fold reduced for combo drugs compared to DOX. In 3D cell cultures, nanoparticles easily penetrated the spheroids, reaching their center. A combination of two drugs was 1.5 times more effective in killing the NCI/ADR and 3.5 times more effective in killing the MCF-7/ADR when compared to DOX only. While further *in vivo* studies are necessary, the authors were able to suggest the good performance of the drug by demonstrating the efficiency of the system in 3D cultures.

Similarly, a pH-responsive hybrid drug delivery system was recently developed by conjugating d-α-tocopheryl polyethylene glycol 1000 succinate (TPGS), a kind of P-gp inhibitor, on the surface of laponite nanodisks to effectively treat DOX-resistant breast cancer cells (MCF-7/ADR) by inhibiting the activity of P-gp-mediated drug efflux and effectively accumulating DOX within cancer cells. *In vivo* results revealed that the formulation outstandingly suppressed MCF-7/ADR tumors with low side effects^[94]^.

A similar system was suggested by Tang *et al.*^[95]^. This team co-loaded curcumin (Cur) and piperine (Pip) into SLNs covered with TPGS and Brij 78 molecules. While Cur is known to possess anticancer properties by downregulating survival pathways, its second mechanism of action involves reducing the drug efflux mechanisms. The latter is also a known mechanism of action of Pip, TGPS, and Brij. Thus, a safe system not involving the usage of chemotherapeutics was suggested for the effective treatment of MDR cancer cells. The system’s performance was tested against the paclitaxel-resistant A2780/Taxol cell line. The studies revealed that Pip, TPGS, and Brij 78 are all able to enhance the therapeutic effect of Cur, which is the strongest for all the molecules combined - reaching over 60% inhibition *in vitro.* While these results seem promising, at their current stage, it is hard to evaluate their actual performance since the biocompatibility with healthy cells was not evaluated, and the ability to selectively accumulate in cancerous tissues was not studied.

Examples of studies that had employed cancer-cell specific targeting as an effective tool to fight chemoresistant cancer using SLN can be found in articles by Garg *et al.*^[96]^, Wang *et al.*^[97]^, and Zheng *et al.*^[98]^ The specific ligand molecules were fucose targeting lectin receptors, hyaluronic acid (HA) targeting CD44 and CD168 (RHAMM) receptors (both lectin and RHAMM receptors are overexpressed in the cellular wall of some cancers), and RGD sequence aimed at integrin α_v_β_3_, which is overexpressed in vessels of various tumors types, respectively.

In the system proposed by Garg *et al.*^[96]^, SNL with a solid core of Gelucire® 50/13 and stearic acid and a solid lipid coating of phospholipid 90 NG and stearyl amine embedded with methotrexate (MTX) were fabricated and coated with fucose (FU) [Figure 6D]. The particles’ average diameter was 174.51 ± 5.1 nm, with an entrapment efficiency of 84.3% ± 1.24%. *In vitro* performance was evaluated against MCF-7 cells, while, *in vivo*, a mice model was used with a pharmaceutically induced skin cancer. The FU coated materials had an improved cytotoxic effect against cancer cells, with the IC_50_ values reduced 5-fold when compared to MTX and 2-fold when compared to uncoated particles, due to increased cellular uptake. *In vivo*, the drug was found to selectively accumulate inside the tumor, inhibiting its growth and granting a 100% survival rate up to 75 days post-treatment.

Moreover, in their study, Wang *et al.*^[97]^ combined materials that specifically targeted HA with pluronic 85 coatings for inhibiting drug efflux. These were tested against PTX-resistant cancers HeLa/PTX cervical and MCF-7/PTX breast cells *in vitro*. The materials easily accumulated inside the cancer cells, reducing their viability by up to 90% (for the highest concentration used). Compared to pure PTX, the IC_50_ for HeLa/PTX was reduced over 40 times. In the *in vivo* model, using HeLa/PTX tumor-bearing mice, the drug was found to be selectively accumulated in the tumor, arresting its growth, indicating that the suggested system might contribute a safe tool able to fight drug-resistant cancers.

Meanwhile, in a study by Zheng *et al.*^[98]^, glycerin monostearate (GMS) was modified with a pH-sensitive liner [adipic acid dihydrazide (HZ)], which was linked to RGD and used to encapsulate DOX. Thus, cancer cell targeting drug carriers that release the drug under the slightly acidic pH of cancerous tissues was made. It was found that the proposed modification enhances the cellular uptake of drugs in MCF-7 and MCF-7/ADR cells, leading to a significant reduction of the IC_50_ values in contrast with pure DOX, an 8-fold reduction in MCF-7 and an 11-fold reduction in MCF-7/ADR cells was observed. Interestingly, the IC_50_ value of the carrier was similar regardless of cell type, indicating that it is very efficient in overcoming drug resistance. In mice bearing MCF-7/ADR xenograft tumors, tumor growth was significantly arrested when compared to pure DOX treatment, which was ineffective. No changes in body weight and non-significant accumulation of the drug carrier throughout the vital organs indicates that the carrier improves the efficacy while reducing the negative side-effects of a DOX-based treatment.

#### Summary

In short, various lipid nanocarriers have a high potential to become the new, standard strategy to treat chemoresistant cancer cells. The specific characteristic of these nanocarriers includes biocompatibility and selectivity. In particular, liposomes can increase the therapeutic efficacy of anti-cancer agents, while SLN in combination with surfactants, contributes to overcoming the drug efflux. Both types of lipid nanocarriers can be fairly easily modified with additional functionalities through ligand attachment, PEGylation, or the introduction of stimuli sensitive components. All of these features contribute to the fact that the lipid nanocarriers are currently one of the few nanocarriers cleared for clinical use (e.g., Abraxane, Taxotere). Still, as for polymeric or viral nanocarriers, the employment of theranostic approaches is elusive and requires multistep modifications, which can reduce the material’s biocompatibility or treatment efficiency.

### Metal-based nanocarriers

#### Gold nanoparticles

Undoubtedly, gold nanoparticles (AuNPs) are among the most widely employed nanosystems for these applications due to their high surface-to-volume ratio, hydrophilicity, and functionality, combined with high *in vivo* stability and a large surface area available for the attachment of materials such as antibodies.

Recently, Pedrosa *et al.*^[99]^ developed multifunctional AuNPs employed as vehicles for the delivery of the Zn(II)-based coordination compound [Zn(DION)2]Cl2 (ZnD) to increase the toxicity of the drug towards DOX-resistant colorectal carcinoma. Selective targeting was achieved through cetuximab which interacts with the epidermal growth factor receptor, typically overexpressed in cancer cells. The authors reported that the nanostructure could significantly inhibit cell proliferation and trigger the death of resistant tumor cells *in vivo*.

Similarly, Rathinaraj *et al.*^[100]^ developed a folate-gold-bilirubin (FGB) nanoconjugate used to efficiently reverse multidrug-resistance in P-expressing KB-ChR-8-5 cells. The FGB nanoconjugate was reported to elicit a stronger inhibition of the cells when compared to a treatment based on either bilirubin or AuNPs alone. The nanoconjugate was able to induce tumor suppression, ROS overexpression, DNA strand breaks, and apoptotic morphological changes in the cells, as tested in a mouse model.

Trastuzumab (Tmab), a monoclonal antibody that targets the human epidermal growth factor receptor 2 (HER2), is another good example of a component that has seen an increased resistance over time, as no current HER2-targeted therapeutic agent is effective against Tmab-resistant gastric cancer. Recently, HER2-targeted AuNPs were produced by successfully conjugating Tmab onto the surface of the NPs. After synthesis, the therapeutic effect of the AuNPs was tested towards HER2-positive Tmab-resistant (MKN7) or Tmab-sensitive (NCI-N87) gastric cancer cell lines, both *in vitro* and *in vivo* via intratumoral injection of the NPs. The anticancer effect was achieved via autophagy of the subcutaneous tumors^[101]^.

Deng *et al.*^[102]^ developed multifunctional AuNPs to simultaneously co-deliver three anticancer agents: AS1411, DOX, and anti-221, which are commonly used to enhance leukemia treatment efficacy. The NPs significantly inhibited the proliferation of the cells by inducing apoptosis, in a process associated with marked downregulation of the short RNA molecule miR-221 and the enzyme DNMT1. Besides, primary blasts obtained from the leukemia patients experiencing chemoresistant relapse were exposed to these NPs, becoming sensitized to DOX.

Pancreatic ductal adenocarcinoma (PDAC) has become one of the deadliest solid cancers found in patients worldwide. A recent report showed that 20 nm diameter AuNPs could sensitize pancreatic cancer cells to the anticancer drug gemcitabine through inhibiting migration and colony-forming ability of pancreatic cancer cells^[103]^.

Similarly, it is well-known that HSP90 (a chaperone protein that assists other proteins to fold properly) inhibitors have the potential to treat many types of cancer due to the dependence of tumor cells on HSP90 for cell growth. Researchers found that Cullin-5 (Cul5) E3 ubiquitin ligase is needed for HSP90 inhibitors to induce cell death via protein degradation. As such, Talamantez-Lyburn *et al.*^[104]^ developed a method for the delivery of Cul5 DNA to cells employing AuNPs. The nanocarriers were able to increase the sensitivity of Cul5 deficient AU565 cells, providing evidence that AuNPs-assisted delivery of DNA can easily lead to the sensitization of drug-resistant tumor cells.

#### Silver nanoparticles

Silver nanoparticles (AgNP) have been extensively used in the treatment of MDR cancer, with a great dependency of the efficacy on the size of the nanostructures. Still, there is an important window for research that would allow targeting some of the deadliest drug-resistant tumors.

As such, a relevant example was shown by Gopisetty *et al.*^[105]^, who decided to examine AgNPs size-dependent cellular features in MCF-7/KCR cells. They reported that 75 nm AgNPs were more efficient in inhibiting the P-glycoprotein (Pgp) efflux activity in the MCF-7/KCR cells (thus potentiating the apoptotic effect of DOX) than the 5 nm nanoparticles. Meanwhile, the 5 nm AgNPs were more effective ROS producers, most likely due to a higher surface-to-volume ratio. It was observed that the 75 nm AgNPs were able to affect the endoplasmic reticulum calcium stores, thus causing the ER stress, and decreasing the plasma membrane positioning of Pgp. This was regarded as a determining mechanism of Pgp inhibition, which was proven effective in sensitizing the multi-drug resistant breast adenocarcinoma towards DOX treatment. Therefore, the 75 nm AgNPs can be used as powerful inhibitors of Pgp function and are promising agents for sensitizing multidrug-resistant cancers to anticancer drugs.

In a similar study, AgNPs were used to sensitize the A2780-Pt cells towards Pt treatment. Cur-coated AgNPs were biologically synthesized. Synergic cellular effects of AgNPs and Pt on the cells were assessed. Results showed that 8 and 62 µg/mL of AgNPs and Pt led to 50% cell death in 48 h^[106]^. Similarly, AgNPs were shown to exert an inhibitory action on the efflux activity of MDR cancer cells, when the synergistic interactions of AgNPs with six different antineoplastic agents on drug-resistant cells were tested^[107]^.

Overall, the use of metal nanoparticles in the treatment of drug-resistant cancers has been widely researched. The surface areas of metal nanoparticles, such as gold and silver nanoparticles, allow for the attachment of bioactive molecules, e.g., antibodies, that help to sensitize the cancer cells,. Furthermore, other chemical properties, such as hydrophilicity and stability, also contribute to effective drug delivery. However, it has been proven that the viability of metal nanoparticles is highly dependent on their size and, to some extent also shape. Thus, there is much more research to be done to improve metal nanoparticles for more effective anticancer treatment.

#### Magnetic nanocarriers

One of the biggest advantages of magnetic nanoparticles (MNPs) - compared with non-magnetic materials - is the possibility to be controlled by an external magnetic field. In the case of nanocarriers, this feature may support particle guidance, uptake, and accumulation at intended destinations, especially in combination with another targeting agent. For example, Wang *et al.*^[108]^ modified yolk-shell Fe3O4@MgSiO3 nanoparticles with polyethylene glycol conjugated with folic acid (FA), which binds folate receptors overexpressed in various cancerous cells^[109]^. An *in vitro* study on drug-resistant GEP-G2/MDR cells confirmed the internalization of these NPs via the protein-independent endocytosis pathway that obviated mechanisms typically associated with a DOX resistance. Moreover, synergism between FA and magnet-assisted targeting resulted in increased accumulation of the particles in the Hep-G2/MDR tumor xenografts in mice models and effectively inhibited tumor growth.

In turn, Cho *et al.*^[110]^ developed magnetic tandem apoptosis triggers (m-TATs), consisting of the magnetic core, targeting monoclonal antibodies, and DOX. Exposure to a static, low-gradient magnetic field facilitated cellular internalization of particles and simultaneously induced their clustering that activated the extrinsic apoptosis cascade process. The synergism of action between m-TAT components resulted in absolute mortality of the DLD-1/ADR colon cancer cell line *in vitro* and complete retraction of the tumor *in vivo* in the xenograft mice model. Instead of using whole antibodies, Truffi *et al.*^[111]^ modified MNPs with multiple half chains of anti-HER2 monoclonal antibodies, Tmab. The potential of these MNPs was tested on various cells, including Tmab-resistant BT474TR and JIM1-4 cells. The particle concentration of 0.2 μg mL-1 was enough to accumulate in 100% and 40% of BT474TR and JIM1-4 cells, respectively. However, the additive effect on Tmab-conjugated particles and DOX on cell viability was observed only in BT474TR cells.

Cell-nanoparticle interactions can also be enhanced by the modification of MNPs with peptides. Miller-Kleinhenz *et al.*^[112]^ developed a peptide with an ability to bind and inhibit simultaneously the Wnt-related LRP5/6 and urokinase plasminogen activator (uPAR) receptors, which was subsequently conjugated with DOX-modified MNPs. This strategy limited cell invasion in MDA-MB-231 via newly recognized mechanisms associated with Axin and β-catenin regulation but did not affect cell viability *in vitro*. An *in vivo* study on the orthotopic human chemo-resistant breast cancer patient-derived xenograft mouse model showed combinatorial effects of chemotherapy and inhibition of the Wnt/beta-catenin signaling pathways, resulting in a significantly reduced number of proliferating cells and minimized average tumor volume.

Furthermore, Liu *et al*.^[113]^ and Weng *et al*.^[114]^ exploited cell-penetrating peptides to deliver Ag-Fe3O4 with DOX and Fe3O4 loaded with Pt, respectively. The proposed NPs were characterized not only by an enhanced internalization but also showed unexpected ability - mediated by ROS produced via the Fenton reaction - to reverse drug-resistance in HNE-1/DDP and CNE-2 cells. The high toxicity of ROS was also employed by Ma *et al.*^[115]^ to enhance the anticancer activity of the Pt prodrug conjugated with FePt NPs - iron. In this study, the release of ROS from the NPs induced mitochondria dysfunction, activating the apoptotic cascade and sensitizing drug-resistant ACP cells to Pt. The magnetic field applied *in vivo* (tumor-bearing mice model) supported the accumulation of the particles in the tumor, contributing to significantly increased ROS levels and greater inhibition of tumor relative volume after 14 days, compared to free Pt.

Enhanced ROS production in cancer cells was also achieved by loading multi-core MNPs with an artemisinin derivative as proposed by Guo *et al.*^[116]^. In this study, the dose-dependent toxicity of the dihydroartemisinin-MNPs complex, superior to those observed for DOX, was proven against both MCF-7 and MCF-7/ADR cells. This was assigned to the release of ferrous ions from MNPs under acidic conditions, which further catalyzed the DHA-related ROS production. In contrast, Yen *et al.*^[117]^ conjugated MNPs with catalase - an enzyme converting ROS to molecular oxygen - to mitigate hypoxic environments in tumors as insufficient oxygen supply activates the production of hypoxia-inducible factors participating in the development of multidrug resistance. Catalase-modified MNPs not only maintained the pH-dependent profile of the enzyme activity, prolonging it 3-fold, but also showed significantly higher toxicity in combination with PTX towards drug-resistant 4T1 cells compared to both free PTX and catalase+PTX treatments.

Many phenolic compounds, famous for their anti-oxidant and anti-inflammatory properties, also showed anti-cancer activity and thus can be used as chemosensitizers^[118]^. Among various phenolic compounds, Cur is one of the most widely exploited, acting as a chemodepressor for co-delivering cytostatics using magnetic nanocarriers^[119-122]^. Rastegar *et al.*^[123]^ developed MNPs modified with hydroxyapatite and β-cyclodextrin, which provided effective encapsulation of DOX and Cur that resulted in significantly reduced cell viability of MCF-7 and MCF-7/ADR cells, compared to free DOX. Even though the magnetic guidance facilitated the accumulation of these particles at the targeted site in 4T1 tumor-bearing mice, the substantial impact of co-delivery on relative tumor volume was not observed in this study. However, the P-gp expression analysis confirmed remarkable Cur-mediated inhibition of the efflux pump.

Quercetin (QUR) was also used to overcome drug-resistance in cancerous cells. Daglioglu^[124]^ developed pH-sensitive MNPs conjugated simultaneously with QUR and DOX that showed an ability to significantly increase the effect of DOX on A546/DOX by augmentation of the G2/M-phase cell cycle arrest leading to reduced cell proliferation and increased apoptosis^[124]^. Besides, this nanosystem showed a limited impact on BEAS-2B cells, which proved that this nanosystem has limited cytotoxicity towards non-cancerous cells.

MNPs were also used to load different types of nanocarriers to enable their control via the magnetic field. Wang *et al.*^[125]^ reported mesoporous silica-based NPs loaded with MNPs for dual chemo/photodynamic therapy, which effectively reversed resistance and induced apoptosis in cancer cells in a dose-dependent manner. The exposure to a magnetic field increased the cellular uptake of the NPs in drug-resistant cells, as well as significantly enhanced their retention in the tumor *in vivo*, which contributed to the prolonged mean survival duration of MCF-7/ADR tumor-bearing mice. Additionally, in a study by Wang *et al.*^[119]^, loading of polymeric particles, made of biotin-poly(ethylene glycol)-poly(Cur-dithio dipropionic acid), with MNPs contributed to significant enhancement in the cellular uptake of the nanosystem by MCF-7/ADR cells upon the magnetic field, which resulted in a 2-fold higher resistance reversion index against cancerous cells, as compared to the non-magnetic-assisted treatment.

Overall, MNPs represent viable methods of the treatment for chemoresistant cancers because they can be controlled by external magnetic fields, which can assist uptake and accumulation of nanocarriers at intended destinations. Many studies have been done to determine the function of MNPs in the treatment of chemo/drug-resistant cancers. MNPs present modified ROS that aid in the reversal of drug resistance in cancer. However, MNPs can present a viability problem with a certain cell line. Thus, more consistent research must be performed with MNPs.

### Carbon-based nanocarriers

**Figure 7:**
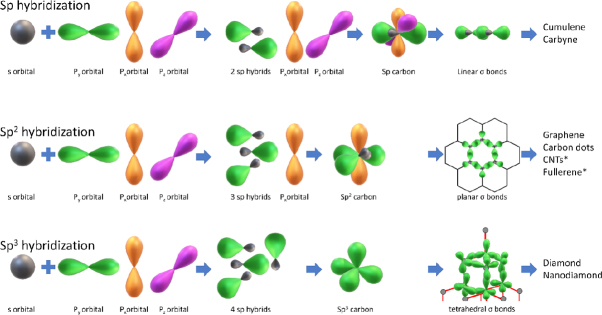
Schematic representation of various hybridizations of carbon, together with their corresponding allotropes. Note, for simplification, CNTs and Fullerenes are presented as sp^2^ carbons. *Denotes transitional hybridization between 2-3

Carbon nanomaterials (CNMs) are reported to produce safe and effective drug delivery systems for targeting therapy-immune cancer cells. CNMs include nanodiamonds (NDs) in sp^3^ hybridization, graphene/graphene oxides (GO), and carbon quantum dots (CD) in sp^2^ hybridization, carbon nanotubes (CNTs), and fullerenes in sp^2-3^ hybridization^[126,127]^, the atomic structure of which is depicted in Figure 7.

**Figure 8:**
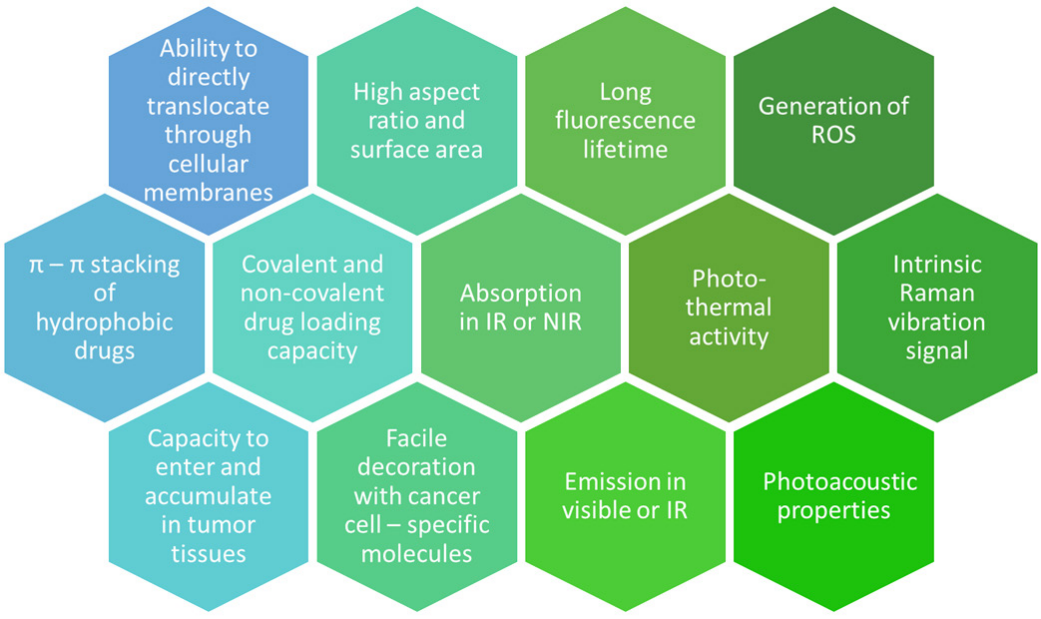
Selected properties of carbon-based nanomaterials that constitute for their popularity in the field of nanomedicine-based anticancer therapy^[141,144,155,]^

Among these, graphene, CNTs, and carbon dots are the most commonly used, and this is due to two unique qualities. Firstly, they possess a large share of delocalized π bonds able to bond hydrophobic drugs by π stacking^[128,129]^. Secondly, they have unique optical properties, such as near-infrared absorption and emission, which allow their usage for imaging and photothermal therapy. Other favorable qualities shared for some CNMs are listed in Figure 8. These properties allow for fabricating multifunctional delivery systems with the possibility of loading more than one cargo onto a single molecule and, at the same time, that enable real-time tracking of NMs distribution by fluorescence, and allow for photothermal (PTT) or photodynamic therapy (PDT, as a photosensitizer and photosensitizer carrier) for an effective theragnostic approach^[130,131]^.

**Figure 9:**
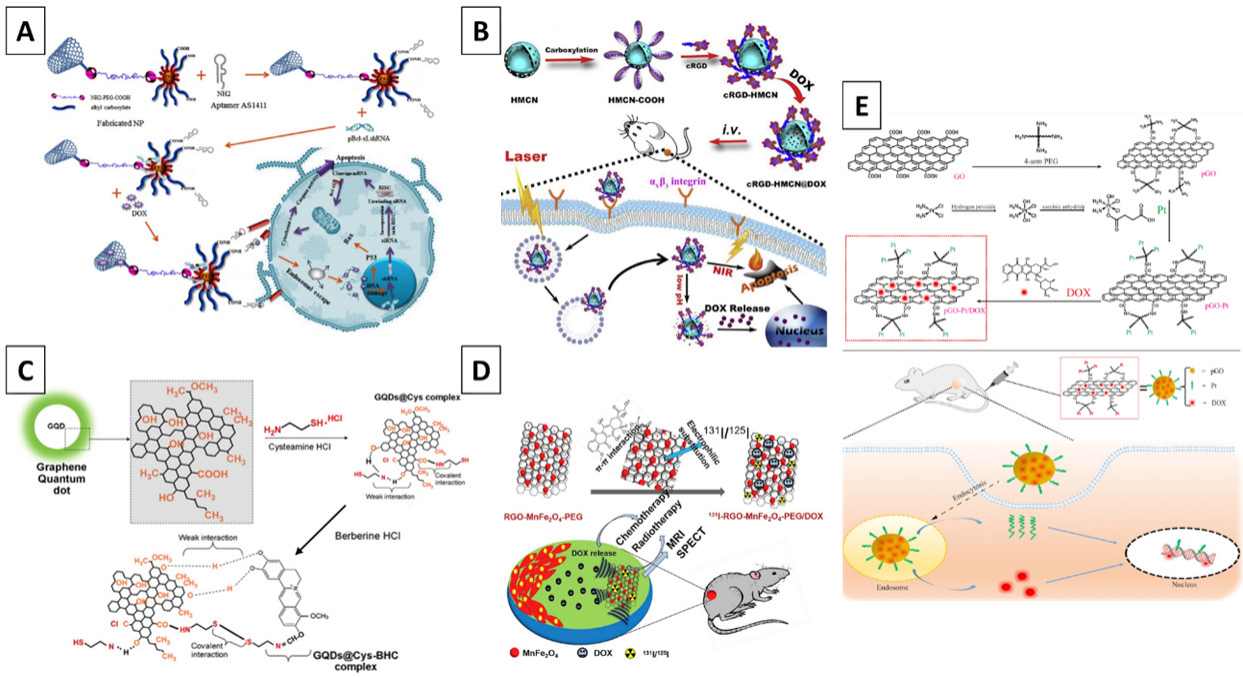
Examples of strategies used to bond more than one bioactive molecule to the CNMs. Covalent bonding of polyethylenimine and aptamers to the surface of PEG-modified CNTs, combined with non-covalent π-π stacking of DOX^[138]^ (A); covalent bonding of cRGD combined with the loading of DOX into the CNMs’ cavity for selective targeting of cancer cells^[135]^ (B); combination of covalent and non-covalent binding of a single drug for the controllable release from graphene quantum dots^[167]^ (C); manganese ferrite grown on the surface of graphene oxide, followed by modification with a radioisotope via electrophilic substitution and π-π stacking of DOX^[143]^ (D); two anticancer drugs, bonded to the surface of graphene via covalent (Pt) and non-covalent (DOX) interactions for an enhanced anticancer effect^[142]^ (E)

The possibility of loading multiple cargos arises from the CNMs’ unique intrinsic properties; cargo can be filled inside the material’s cavity (“endohedral filling”). Also, other factors, such as interactions with the CNMs’ surface (electrostatic, click chemistry, covalent^[132,133]^) and π-π stacking, contribute to the transport of additional materials^[134]^. By this means, due to an extremely high surface area, high amounts of various molecules could be bonded to the same particle. These cargos can be quickly released in the acidic environments of endosomes and lysosomes of cancer cells that can cleave the drug-CNMs interactions^[135]^. Examples of this approach are visualized in Figure 9, and these can be divided by the nature of the bonded species:

1. Hydrophobic drugs (such as DOX, SN38, and PTX) can be bonded by the π-π stacking mechanism for improved dispersion and facilitated release of those drugs^[136-138]^;
2. Different targeting molecules for selective delivery to cancer cells can be attached via click chemistry or covalent modification. Some examples are: (a) ferritin^[139,140]^ and transferrin^141^, both of which bind to cells via the transferrin receptor 1, overexpression in cancerous cells; (b) iRGD or cRGD for targeting the avb3-integrin of tumor endothelium; and (c) candesartan (CD) for targeting AT1R in lung cancer cells^[141,142]^;
3. Biological compounds, such as peptides, proteins, nucleic acid, *etc*. are attached via covalent interactions, click chemistry, or electrostatic attraction, enabling improved solubility, biocompatibility, and increased protection from uncontrolled cleavage and degradation^[136,137]^;
4. Magnetic and ferromagnetic particles can be entrapped within the materials cavity or bonded to their surface for magnetic field-assisted selective delivery and enhanced contrast for magnetic resonance imaging^[143]^.

CNMs can have various sizes that can be comparable to natural compounds: proteins (1-100nm) and DNA (2-3nm wide)^[144]^. They can enter the cells through passive diffusion (also referred to as needle-like penetration, mainly reported for CNTs)^[145]^ or various adenosine triphosphate (ATP)-dependent uptake mechanisms (dynamin-dependent)^[146]^. The leading uptake mechanism depends on the CNMs’ physicochemical properties and the type of the cell^[145]^.

Due to the high popularity of CNMs in the cancer theragnostic, many articles on the matter can be found, and additional background information can be found in these excellent articles^[130,141,144,147-155]^. Additionally, information about how CNMs’ intrinsic properties can serve in theragnostic approaches [PTT and PDT, optical imaging, and cargo delivery systems (including stimuli-responsive and cancer cell-selective)] and how to obtain biocompatible CNMs can be found in these sources. By combining different cancer-targeting strategies and facilitating selective intake and accumulation in cancer cells, CNMs can be regarded as effective tools for fighting chemoresistance in cancer cells. Most notably, it has been reported that CNMs-based PTT can reverse the resistance of cancer cells to a certain drug and target cancer stem cells responsible for remission^[156]^. In the following sections, examples of some of the most interesting and recent approaches in targeting cancer cells using various CNMs based cargo-delivery systems are given. These are grouped by the type of CNM.

#### Carbon nanoparticles

Carbon nanoparticles are spherical nanomaterials, typically made up of amorphous carbon, with varying amounts of graphitic planes. The presence of the latter allows for π-π stacking of hydrophobic drugs and grants the materials with excitation dependence - fluorescence, typically in the NIR-I window^[157]^. Most often, these materials will possess a hollow core, into which a magnetic particle can be loaded^[158]^, and a mesoporous structure, useful to load bioactive molecules^[135]^. Oxidation can be used to introduce functional groups for enhanced dispersion and covalent interactions with drugs^[157]^.

Depending on the synthesis method, the sizes of the CNPs can range from a few nanometers to a few hundred nanometers, enabling various internalization pathways. In cancer drug delivery, the CNPs are most typically loaded with hydrophobic drugs, and the therapeutic effect can be boosted by gene or thermal therapy. In a study by Zhao *et al.*^[159]^, the morphology dependent performance of CNPs was tested, and it was found that spherical, rough, and hollow particles are superior in employing a combined gene/PT/chemotherapy able to kill over 90% of a mice analog of metastatic, chemoresistant stage IV breast cancer cells, both *in vitro* and *in vivo.* Remarkably, the authors had managed to fabricate materials with the NIR-II fluorescence window for effective PT. This region is favorable as it yields a more effective emission, even in deep tissue, due to reduced scattering^[144]^.

In a different approach, studied by Fan *et al*.^[139]^, the CNPs were found to produce a cancer cell-killing effect without the usage of an external bioactive molecule. Conjugation with hollow human H-ferritin NPs yielded a selective and effective material that entered the cells via clathrin-dependent endocytosis, localized in lysosomes, and caused ROS burst and hypoxia. Intravenous injection *in vivo* resulted in completely arrested hepatocellular carcinoma and colon cancer tumor growth at day 5, with mortality reduced to zero and a complete recovery reported in 40% cases, and no damage to vital organs observed. The particles were safely cleared through urine and feces. These remarkable results should be treated as a guide for the future development of effective, nanomaterial-guided cancer eradication. Because no drug was used, it should be hypothesized that this strategy would be effective in treating chemoresistant cancers. However, further evaluation should be performed to confirm such supposition.

Other teams have also reported successful and selective eradication of chemoresistant, metastatic cancers *in vivo*, by enabling the delivery of DOX and CPT^[135,160-162]^. In a study by Li *et al.*^[161]^, mesoporous carbon nanospheres were loaded with DOX and coated with PEG - poly(Cur-dithiodipropionic acid) (PEG-PCDA). The latter was used to grant the materials with improved dispersibility and provide them with chemosensitizing quality. Upon entering the cells, glutathione of the cytoplasm cleaved the bonds in the (PEG-PCDA), leading to Cur release, which increases the drug accumulation in the cancer cells. After the shell of the CNM was removed, the pH-sensitive release of DOX was able to take place. The obtained system was used to kill chemoresistant MCF-7/ADR cells effectively. Still, further studies are needed to evaluate whether or not the system can be safely used - *in vitro* studies to establish cytotoxicity against normal and healthy cells, followed by *in vivo* evaluation to determine the long-term effect and fate of the CNMs in the living organism.

#### Carbon dots

The term carbon dots (CDs) most typically refers to small fragments (usually a few nm) of graphene planes, cut down via chemical or physical treatments. To avoid agglomeration, oxygen atoms are introduced into the structure. CDs combine features characteristic of graphene - NIR fluorescence, PT effect - with simpler internalization and the ability to be eliminated from the body via urinary clearance.

In the literature that regards nanomedicines against cancer, CDs are typically used for better tumor visualization, in PTT, and for fabricating pH-sensitive drug delivery systems^[163,164]^. Selective accumulation is achieved by the EPR effect^[165]^ or through specific modification, e.g., by folic acid^[166]^. In the study against stage IV metastatic breast cancer cells, CDs were able to deliver BHC drugs inside the cells, inducing apoptosis in 99% of the culture and granting the ability to visualize the cells via fluorescence^[167]^. In a different study, Feng *et al.*^[164]^ modified the CDs with Pt and DOX using pH cleavable covalent bonds. The materials were easily internalized by Pt-resistant human ovarian cancer cells, enabling fluorescent imaging. Under the acidic pH of cancer cells, a controllable release of drugs resulted in the efficient killing of cancer cells at significantly lower concentrations than the Pt alone. Unfortunately, an effect on the healthy cells was not evaluated, and the materials were not subjected to extensive *in vitro* or *in vivo* evaluation. A more comprehensive evaluation was done by Sui *et al.*^[168]^, who proposed a drug delivery system able to selectively attack cancer cells and sensitize Pt resistant cancer for effective chemotherapy treatment. No adverse effect on healthy cells was observed, both *in vitro* and *in vivo*.

#### Nanodiamonds

Nanodiamonds are nanoscaled materials (typically up to tens of nm), composed mainly of carbon atoms in the sp^3^ hybridization (tetrahedral). For such materials to exist as stand-alone structures, the outermost atoms need to be capped. This is done either by heteroatoms or by carbon atoms in different hybridizations. As such, NDs are open for functionalization strategies that can anchor different molecules. Additionally, the existence of specific defects in the structure can grant the NDs with photoluminescence, enabling PTT. These features are highly desired in nanomedicine^[144,169,170]^. In a study by Yu *et al.*^[171]^, the performance of NDs in delivering PTX into chemoresistant cells was compared with a commercially available NPs-based system, Abraxane. It was found that the particles readily accumulated in lysosomes, and the efficiency of killing cancer cells was significantly improved as compared to Abraxane (71% cells were killed as compared to 40% upon 48 h culture). This clearly indicated the superior performance of the NDs in this field, most likely attributed to better uptake and pH-sensitive drug release.

NDs have also been reported to successfully deliver drugs used to fight chemoresistance in cancer cells by blocking the P-glycoprotein from pumping-out, which is over-expressed in multi-drug resistant cancer cells and participates in the clearance of some of the anticancer drugs, such as DOX or Pt. In a study by Zhu *et al.*^[172]^, a double drug loading of NDs with a physisorbed malaridine (MAL) and DOX, followed by covering with folate-DOX-PEG, was done. The resultant material was easily dispersed, readily entered the cells, and revealed a pH-responsive cargo release. The presence of MAL increased the treatment efficiency against chemo-resistant MCF-7/ADR cells by improving the cellular accumulation of DOX. In *in vitro* studies, apoptosis was induced in over 90% of cells, proving the high efficiency of this combinatorial approach. However, further studies need to be done to prove the system’s safety *in vivo*. On the other hand, Lam *et al.*^[173]^ has shown that NDs by themselves can also be used to reduce the P-glycoprotein efflux. By covering the NDs with either GEF or erlotinib (EL), a promising therapeutic effect was achieved.

The efficiency of the anti-cancer treatment can also be increased by introducing specific targeting molecules. This avenue was investigated by Chan *et al.*^[174]^. Selective targeting of cancer cells over healthy cells was achieved by introducing folic acid (FA) moieties. To enhance the performance even further, a mitochondrial localizing sequence (MLS) was used to favor drug accumulation in mitochondria over lysozymes. As a consequence, a superior system that was safe to healthy cells but detrimental to cancer was obtained^[174]^.

In a different study by Li *et al.*^[175]^, NDs were used to fabricate an efficient glioblastoma-targeted treatment. Glioblastoma is a highly malignant brain cancer that has very mortality due to chemoresistance, lack of effective treatments, and the inability of pharmaceutics to cross the blood-brain barrier (BBB). In the study, NDs modified with DOX were taken up by dendritic cells (DC), which were used to deliver a payload through BBB and into glioblastoma cells (GC). As a result, adjuvanticity and antigenicity of the GC cells were stimulated, which, in turn, promoted maturation of DC and initiation of an immune response via T lymphocytes. Thus, the material was suggested as a novel tool able to subvert the glioblastoma immunosuppressive microenvironment and to employ anti-GBM immunotherapy. Thus, a selective accumulation in the brain was achieved, indicating that the therapy should be safe and not affecting vital organs^[175]^. In the follow-up studies, the mechanism of the NDs-related action was attributed to the activation of autophagy^[176,177]^. In a different study by the same team, the NDs-DOX complex was found to suppress the glioblastoma - astrocyte cross-talk, breaking the chain responsible for acquiring resistance towards various therapies by evading apoptosis^[178]^. The same material was also used to evade chemoresistance and reverse cancer-induced immunosuppression in triple-negative breast cancer^[179]^. Thus, the NDs can be suggested as one of the most promising nanomaterials for the treatment of therapy-resistant cancers.

#### Graphene and graphene oxide

Graphene is a 2-D material made up of a single honeycomb lattice. This material has roughly a 2-times higher surface area than CNTs (suitable for drug delivery) and better electrical properties. Fluorescence is observed from the ultraviolet (UV) to near-infrared (NIR). Heat can be emitted upon absorption of light or acoustic waves, enabling PTT or thermoacoustic therapy. Selective imaging with better contrast can be achieved via fluorescence, photothermal, photoacoustic, and Raman scattering effects. Thus, graphene possesses a set of properties, making it suitable to be used in novel anti-cancer therapies. However, one limitation lies in the fact that it contains a high amount of unlocalized π electrons, causing a spontaneous aggregation of graphene planes, reducing the material’s applicability. Hence, its oxidized form - GO^[144]^ - is the most typically employed.

GO has been successfully used in targeting different types of tumors, including metastatic and chemoresistant cancers. In a study by Pei *et al.*^[142]^, the combination of two chemotherapeutic drugs, PTX and DOX, delivered into the cells by GO resulted in an effective eradication of cancers, both *in vitro* and *in vivo*, without adverse side effects. Remarkably, CNMs were able to reduce the size of highly chemoresistant tumors by over 60%. A similar approach of combining more than one drug was also employed by Tiwari *et al.*^[180]^ and Tran *et al.*^[181]^ with similarly promising results.

In a study by Thapa *et al.*^[182]^, DOX was combined with RAPA, an inhibitor of phosphoinositide-3-kinase (PI3K)/Akt/mechanistic target of rapamycin (mTOR) cell survival pathways, one of the many mechanisms of resistance development. By combining the material with PTT, nearly 80% of chemoresistant cancer cells were killed *in vitro.* Certainly, further studies on the safety and *in vivo* efficiency of the system are needed. In different studies, modification with drug targeting molecules, such as lactobionic acid^[183]^, hypericin^[184]^, prostate stem cell antigen (PSCA) monoclonal antibody (mAb_psca_)^[185]^ or hyaluronic acid^[186]^ had resulted in a more selective and effective eradication of cancer, reaching up to 80% killing efficiency via induction of apoptosis. Interestingly, in the study by Guo *et al.*^[185]^, additional modification with magnetic particles allowed for imaging of tumors with high resolution and contrast. The cancer-killing efficiency can be further improved by employing PT^[181,182]^ or radiotherapy^[143]^, with tumor cell viability reduced to up to 90%. Therefore, it can be concluded that effective killing of chemoresistant cancer cells can be achieved by employing GO as a carrier combined with multimodal therapies, such as multiple drug usage, radiotherapy, PTT, and employing cancer-targeting molecules. These therapies sensitize the cells towards further treatment, enabling the effective delivery of higher doses of therapeutic agents into the cancer cells while minimizing the risk of adverse side effects.

#### Carbon nanotubes

Carbon nanotubes (CNTs) are composed of cylindrically rolled-up graphene planes, either single (SWCNTs) or multiple (MWCNTs). The typical diameter of SWCNTs is below 10 nm, which helps facilitate passive transport into the cells; while, at the same time, results in higher cytotoxicity. Meanwhile, MWCNTs with diameters exceeding 15 nm are usually reported to be less toxic^[187]^. In both cases, reduced lengths contribute to reduced toxicity, both *in vitro* and *in vivo*. Regardless of the administration route and hydrophilicity, dispersible materials generally have better applications^[188]^. Depending on the rolling axis, three different conformations of SWCNTs can be obtained, which determine electrical and optical properties, such as arm-chair (metallic), chiral, or zig-zag (semi-conducting). From the cancer drug delivery point of view, semiconducting SWCNTs are beneficial as they present photoluminescence in the NIR-II window upon excitation, enabling imaging of deep tissues with good contrast^[144]^. MWCNTs usually present metallic conductivity and, in some cases, exhibit photoluminescence and photothermal properties^[156,189]^.

As a literature survey reveals, MWCNTs are more often applied in fighting chemoresistant cancer than SWCNTs, which is probably due to higher uniformity of electrical and optical properties and better biocompatibility. With encapsulation efficiency usually exceeding 50%, internalization via endocytosis, and sustained linear drug release upon cellular internalization, these materials seem to be very promising candidates as novel therapies against chemoresistant cancers. In a series of studies by Dong *et al.*^[156,189]^, oxidized MWCNTs were π-π stacked with DOX and modified with TAT-chitosan conjugate to fabricate a biocompatible platform for the sustained release of the drug. Intratumoral administration into mice bearing BEL-7402 xenograft tumors resulted in an effective reduction of tumor size for improved survival rates by combining PTT with drug-related effects. Raza *et al.*^[190]^ had used oxidized MWCNTs to bind two types of bioactive molecules via different bonds. The results indicated that the obtained nanomedicine is biocompatible and able to target cancer cells. This resulted in sensitized chemoresistant MDA-MB-231 cells. In a study by Zhang *et al*.^[191]^, MWCNTs were covalently bonded with gadolinium-conjugated CDs and π-π stacked with DOX, followed by modification with an EGFR antibody. The materials were selectively uptaken by A549 cells and metastatic MDA-MB-231 cells, and exhibited pH and NIR-responsive drug release, with a photothermal effect. In *in vivo* experiments, A549 tumor-bearing mice, subjected to intratumoral injections and PTT, revealed a 100% tumor elimination. Sadly, as *in vivo* studies were not performed on chemoresistant cancers and the intratumoral administration route is not often clinically applied, it is hard to evaluate the actual value and the clinical transferability of the presented data. Certainly, further studies are needed.

Safe and successful usage of SWCNTs in eradicating cancer cells has also been reported. Most notably, in a series of studies by Guven *et al.*^[192,193]^, the ability to fabricate highly selective nanomedicine that can be delivered and released in a controlled matter was found. For example, a highly potent and hydrophobic drug, Pt, with the efficient killing of cancer cells, was created. The accumulation of NMs in cancer cells was attributed to the EPR effect. In a different study by Ghosh *et al*.^[194]^, estradiol positive (EP) cancers were targeted by modifying SWCNTs with 17β-estradiol based amphiphiles, which resulted in the selective killing of ca. 70% of EP cancers. While CNTs are most typically suggested as an efficient tool used to bind hydrophobic drugs for improved administration efficiency, a completely different approach was suggested by Razzazan *et al.*^[195]^. In this study, binding with CNTs was used to improve retention of a hydrophilic drug (GEM), increasing its resistance towards enzymatic attack and improving its plasma half-life. At the same time, efficient killing of cancer cells, such as, MIA PaCa-2, pancreatic cancer cells, which have a significant tendency of developing drug resistance via epithelial-mesenchymal transition, was achieved *in vitro. In vivo*, a selective delivery was proven, with smart, pH-dependent drug release. As a result, tumor growth was significantly arrested, with one case of complete recovery reported.

#### Summary

**Figure 10:**
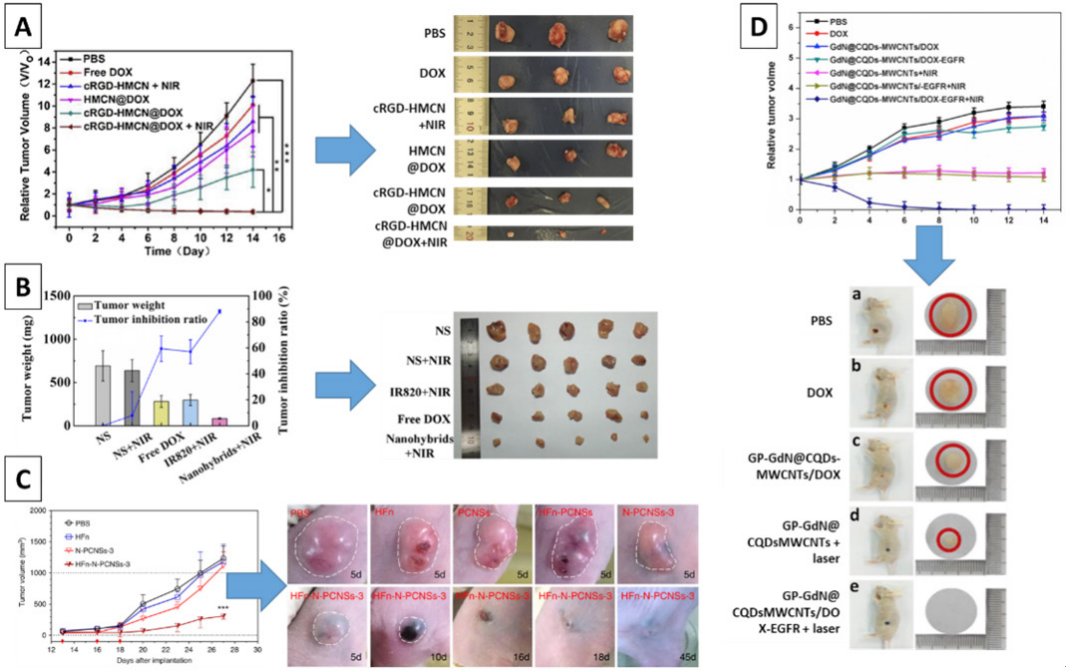
Examples of efficient targeting of tumors *in vivo*, with the aid of CNMs. Size reduction (A and B) and complete tumor eradication (C and D) can be observed. The materials are (A) carbon nanoparticles^[135]^, (B) GO^[183]^, (C) carbon nanozyme^[139]^, and (D) CNTs^[191]^

In summary, carbon-based nanomaterials can be regarded as one of the most promising nanomaterials to be applied in nanomedicine-aided anti-cancer therapy. The ability of these nanomaterials to bind multiple molecules in conjunction with the employment of PTT, PDT, and other complementary techniques has proven to sensitize drug-resistant cancers and provide multiple cell-killing strategies for effective eradication of cancer, both *in vitro* and *in vivo* [Figure 10]. CNMs have also proven the ability to provide cancer-selective vehicles with no detrimental effect observed towards healthy cells or living organisms. The selectivity can be achieved via the EPR effect, functionalization with target specific molecules, or modification with magnetic particles. However, when considering CNMs-based cancer-killing strategies, strict caution needs to be applied - these materials are known to induce varying reactions towards healthy cells and organisms, strictly depending on their physicochemical properties. A broad spectrum from complete biocompatibility to severe toxicity or tumorigenicity is covered. This phenomenon is the main reason for high discrepancies and controversies regarding biomedical usage of CNMs, unlike other materials cited in this study that are, in general, intrinsically biocompatible.

## Conclusion and future remarks

Over the past few decades, many cancers have been reported to show some variable degree of drug resistance to the usual treatments, a reality that is craving for new ways to treat these tumors. There are two categories of factors that are responsible for drug resistance: intrinsic and extrinsic. Drug-resistance in cancer cells prevents the onset of cell death, autophagy, and apoptosis. Thus, new methods are being developed for the treatment of resistant cancer incorporating the use of nanomaterials, as discussed in this review. Nanomaterials can be designed for passive or active targeting of cancer cells, and they can help in transport, delivery, and release therapeutic effects to desired targets. The different types of nanocarriers include polymeric, viral, lipidic, metal-based (including a specific subclass - magnetic), and carbon-based.

While polymeric micelles and colloidal particles are more promising due to their high intrinsic biocompatibility and ability to easily penetrate the cellular membrane, metallic and CNMs show promise because they can provide more cancer-killing mechanisms, including PTT, PDT, and allow for direct visualization, enabling a theranostic approach. VPNs, and their specific subclass, OVs, on the other hand, are a completely different class of NMs, which can easily enter the cancer cells where they can deliver their cargo and/or transfect the cells for efficient tumor eradication, combined with immunity development.

Certainly, one of the main challenges nowadays in designing and successfully employing the novel NMs systems for fighting chemoresistant cancer is to guarantee high cancer-killing efficiency while still maintaining satisfactory systemic biocompatibility of the system *in vivo.* A literature survey presented herein seems to be pointing out that these goals cannot be achieved by using simple, two- or three-component systems. Specifically, a simple drug carrier strategy does not seem to be sufficiently effective. This is because chemoresistant and metastatic cancer is very hard to kill and the remission rate is significant. Hence, one does not only need for the NM-system to selectively target the cancer cells but also there are needs for the system to allow for the employment of additional cancer-killing mechanisms, such as PDT or PTT which can boost the therapeutic effect, additionally sensitizing the cells. Preferably, such a system should also be combined with an ability to directly visualize the cancer cells inside the body for simple, high-resolution tracking of treatment efficiency and cancer response. This approach allows for real-time diagnosis, creating an ability for an alternate treatment as needed.

These goals can be achieved through multiple routes, the simplest idea being polymeric and lipid carriers, filled with metallic or carbon nanoparticles and covered/embedded with specific cancer-targeting molecules. Such a carrier may also contain a drug and/or VPN. However, while the idea is simple, the complexity of the fabrication procedure, a risk of constituent chemical or physical cross-interactions, which can reduce their effectiveness, combined with the relatively large size of the system, significantly limits the practical employment of this approach. For these reasons, utilizing the metal and carbon-based NMs’ intrinsic properties seems to be more promising. This approach needs employing strict fabrication and handling procedures, combined with extensive physicochemical analysis of the carrier, which only then would allow for the obtainment of safe and biocompatible systems. Further functionalization techniques can be tailored and employed to introduce strictly desired drug binding/cancer cell targeting mechanisms. Because these steps can be extremely time-consuming and expensive, they are rarely properly employed, leading to large discrepancies and skepticism of the scientific community in CNMs and metallic particles biomedical applications. Still, as the literature survey suggests, upon careful selection, these NMs can yield results unparalleled with other systems, creating multiple drug binding options, being effective PDT and PTT systems, and allowing for a theranostic approach due to their magnetic and/or fluorescence properties.

Overall, metal-based nanoparticles and carbon-based nanomaterials present extremely viable options for cancer-treatment because of their biocompatibility and stability. However, other nanomaterials are being explored in research that likely will eventually produce highly-valuable therapeutics.

## Declarations

### Availability of data and materials

All data sources could be available to readers on request.

### Copyright

© The Author(s) 2021.
